# Spontaneous Expulsion of the Os Hyoides

**Published:** 1845-03

**Authors:** 


*Spontaneous Expulsion of the Os Hyoides.*—A middle aged woman, of a rachitic constitution, became affected with an enlargement of the glands around the lower jaw, accompanied with a slight cough, and disturbance of the breathing. In spite of treatment these symptoms went on gradually increasing, for three or four years. The sputa became thick and viscid, and were occasionally streaked with blood; the patient was every now and then distressed with attacks of suffocative dyspnœa, and her strength was greatly exhausted by colliquative sweats. The voice was at length completely extinct; and now there was a fixed pain, with an almost continual sense of pricking, in the laryngeal region. The sputa were at this time decidedly purulent, and were often rejected without any effort of expectoration: it was a simple expuition that followed immediately after a lacerating pain felt in the throat.

The condition of the patient had for a length of time appeared quite hopeless, when most unexpectedly—after experiencing more than usual suffering from the cough, dyspnœa and pricking pain in the throat—she expectorated in the midst of a convulsive agitation of the whole body, a firm hard substance, which proved to be a bone of considerable size. On being examined, it proved to be the *os hyoides.* The health of the patient speedily improved, and was ultimately quite restored—five years after the commencement of her first suffering. The bone was examined by several members of the Academy, so that no reasonable doubt as to its nature can be entertained.—*Ibid, from Comptes Rendus.*

